# ENSEAL® Hemorrhoidectomy, a Novel Technique, Versus Conventional Open Method for the Management of Grade III and IV Hemorrhoids

**DOI:** 10.7759/cureus.30834

**Published:** 2022-10-29

**Authors:** Sidra Javed, Adeel Kaiser, Anwar Zeb Khan, Amna Javed, Shabbir Chaudhary, Amna Javed, Muhammad H Shahid

**Affiliations:** 1 Surgery, Lahore General Hospital, Lahore, PAK; 2 Surgery, Sahiwal Medical College, Sahiwal, PAK

**Keywords:** per rectal bleed, hemorrhoids, hemorrhoidectomy, milligan morgan, enseal®

## Abstract

Background

Surgical removal of hemorrhoids is the gold-standard treatment for symptomatic grade III and IV hemorrhoid disease. There are numerous ways the hemorrhoidectomy surgical procedure is done but the most effective and least painful way is still to be elucidated.

Objective

To compare the outcomes of ENSEAL® (Ethicon, Inc., Raritan, USA) versus gold standard Milligan-Morgan hemorrhoidectomy in patients presenting with grade-III and IV hemorrhoids

Materials and methods

After ethical approval, the Randomized Controlled Trial was conducted at the Department of Surgery, Unit III, Lahore General Hospital, Lahore, Pakistan, between January 2020 and January 2022. In this study, 140 patients who met the inclusion criteria were recruited after informed consent. Patients were split randomly into two equal groups using a lottery technique. In group A, hemorrhoidectomy was carried out with ENSEAL®, whereas in group B, open hemorrhoidectomy was performed by the Milligan-Morgan method. the surgery duration and blood loss were noted. After the operation, patients were transferred to and discharged from the post-anesthesia recovery room. Patients were further followed up for pain scores after 24 hours. Data was analyzed by using Statistical Package for Social Sciences (SPSS) v25 (IBM Corp., Armonk, USA). Data was categorized for age, gender, body mass index (BMI), degree of hemorrhoids, and duration of hemorrhoids. A p-value <0.05 was considered significant.

Results

140 patients were included in this study. Group A patients underwent ENSEAL® hemorrhoidectomy, and group B was formed from those who underwent the Milligan-Morgan procedure. In group A, there were 41 (58.5%) males and 29 (41.4%) females, while in group B, there were 43 (61.4%) males and 27 (38.5%) females. The mean age of group A patients was 49.97 ± 7.36 years and 43.2 ± 8.01 years in group B. In group A, the mean operative time was 20.87 ± 3.05 min, while 27.10 ± 3.42 min in group B, which is statistically significant with a p-value of <0.001. In group A, mean blood loss was 9.79 ± 2.87 ml, while 13.36 ± 3.73 ml in group B, which is statistically significant with a p-value of <0.001. In group A, the mean pain score was 2.7 ± 1.08, while 3.34 ± 1.16 in group B, which is statistically significant with a p-value of <0.001.

Conclusion

When considering the length of the procedure and blood loss, ENSEAL® hemorrhoidectomy has been determined to be an effective treatment that the patients tolerated well. Therefore, ENSEAL® hemorrhoidectomy can be a safe and efficient alternative to conventional treatment for hemorrhoids that are causing symptoms.

## Introduction

Hemorrhoidal illness is one of the most prevalent benign anorectal conditions, characterized by bleeding and prolapse. According to research, the prevalence among adults is very high [1]. The global prevalence of hemorrhoids is reported to be 4.4%, with a peak incidence between the ages of 45 and 65. Moreover, 50% of the over-50 population has had hemorrhoid-related issues. Hemorrhoids may resemble various anorectal disorders, including skin tags, abscesses, fissures, polyps, inflammatory bowel disease (IBD), and anorectal cancers [2].

Hemorrhoids are pathological alterations to the anal cushions, a natural component of the anal canal that facilitates stool evacuation and fine-tuning anal continence. These pathological alterations include a rupture of the connective tissue supporting the cushions, resulting in an expansion of the vascular plexus. The pathophysiology of hemorrhoids describes the symptoms associated with the problem, including bleeding, swelling, prolapse, leakage owing to the disturbance of the fine-tuning of continence, and irritation of the perianal skin. The most frequent manifestation of hemorrhoids is rectal bleeding, which is painless and occurs during or shortly after defecation. Extreme symptoms may include thrombosis causing discomfort [3].

No one knows the exact mechanism that causes hemorrhoids. Since Burkitt and Graham-Stewart's study [4], published in the 1970s, it has been assumed that a diet low in fiber and constipation contributes to the occurrence of hemorrhoids. According to the most recent hypothesis, constipation results in persistent straining and firm stools, which induce degradation of the anal canal supporting tissue and shift the anal cushions distally. The concept that hemorrhoids and constipation have different epidemiologic factors, such as age, sex, ethnicity, and socioeconomic status, raises doubts about constipation as a contributing cause. According to a survey conducted at the Milwaukee VA Medical Center [5], diarrhea, not constipation, is associated with hemorrhoids. A study suggests that the comorbid conditions associated with hemorrhoids were all diarrheal (colitis, malabsorption, intestinal bypass, and chronic pancreatitis), not constipation [6].

Due to the unpleasant aspect of hemorrhoidectomy and the significant frequency of hemorrhoidal disease, there is much interest in outpatient therapies for hemorrhoids. There has been modest success with rubber-band ligation, injectable sclerotherapy, infrared photocoagulation, and cryotherapy, but the outcomes are inferior to surgery. Excision and ligation of the hemorrhoidal pedicle, with or without defect closure, is the standard surgical approach for prolapsed hemorrhoids. As established in 1937, Milligan-Morgan haemorrhoidectomy continues to be the traditional surgical method. Over the last two decades, a significant amount of study has been done to decrease postoperative pain after a wide range of surgical operations. Variations include open, partially open, and closed incisions, routine lateral internal anal sphincterotomy, and the use of stapling devices [7].

The hemorrhoidal disease of grades III and IV (Goligher classification) improves well with surgical therapy. Open hemorrhoidectomy (OH) remains the gold standard procedure. However, it is accompanied by substantial postoperative discomfort and a modest risk of anal sphincter complex damage [8]. Changing one's lifestyle and administering phlebotonics constitute the initial treatment for hemorrhoidal illness. After the failure of conservative care, interventional therapies are used to treat hemorrhoidal disease [9].

Over the past decade, minimally invasive surgical techniques have become standard in all surgical fields, including general surgery, gynecology, and urology. Ultrasonic Coagulating Shears (UCS) and Electrothermal Bipolar Vessel Sealers (EBVS) are often employed instruments for dissection, hemostasis, and ligation of the pedicle. In contrast to traditional monopolar diathermy, which can only cut tissue with minimal coagulation, these devices can cut and coagulate tissue and vasculature [10].

Postoperative discomfort is one of the patient's worries, forcing people to decline the operation. Multiple factors contribute to postoperative discomfort, including the anesthetic approach, hemorrhoidectomy procedure, postsurgical inflammation, secondary infection, and sphincter spasm. Anal sphincter spasm is crucial to post-hemorrhoidectomy pain [11].

ENSEAL® (Ethicon, Inc., Raritan, USA) is one of the newest electrosurgical bipolar devices that are currently available. It utilizes nanotechnology to synchronously seal and cut the tissues along with the coagulation of vessels up to 7 mm in diameter. ENSEAL® technology incorporates an electrode comprising nm-size conductive particles enclosed in temperature-sensitive polymers that maintain the temperature of the tissue at 100°C [10].

The aim of this study is to compare the outcomes of ENSEAL® versus gold standard Milligan-Morgan haemorrhoidectomy in patients presenting with grade-III and IV hemorrhoids.

## Materials and methods

The study was performed by the Department of Surgery, General Hospital, Lahore. The study was conducted from January 2020 to January 2022. The study design was an unblinded Randomized Controlled Trial, and participants were split randomly into two equal groups using the lottery technique. The sample size of 140 (70 in each group) was included by using Cochran’s sample size formula.

Inclusion criteria

All patients aged 20 to 60 years of either gender with grade III and grade IV hemorrhoids were included in the study.

Exclusion criteria

The exclusion criteria were: (1) Patients with infected and thrombosed hemorrhoids (on clinical examination); (2) Patients who had previous anorectal surgery, having anal fissures and fistula; and (3) Patients with uncontrolled diabetes mellitus (blood sugar random (BSR) >200 mg/dl), blood pressure (BP) >140/90 mmHg, International Normalised Ratio (INR) >2.

Data collection procedure

Following permission from the hospital's ethics committee (Article/Research Review Committee Post Graduate Medical Institute, Ameer-ud-Din Medical College, Lahore General Hospital, Lahore - IRB No. 28A-15-20), the surgical department of General Hospital, Lahore included 140 patients with Grade III or Grade IV hemorrhoids who fit the study's inclusion criteria.

Each patient's consent was acquired after complete understanding. The demographic details (name, age, gender, BMI, grades, and duration of symptoms) were collected. Patients were randomized into two categories using a lottery system. Group A patients were treated with ENSEAL®, whereas group B patients had Milligan-Morgan hemorrhoidectomy.

Patients were placed in a lithotomy position for surgery under spinal anesthesia. In the Milligan-Morgan technique, a V-shaped incision was made at the anoderm and the haemorrhoidal pedicle was ligated with a 2/0 vicryl suture. 

In ENSEAL®, hemorrhoidal tissue with pedicle was sealed and cut by the calculated arrangement of pressure and radiofrequency. An anal pack was placed in both techniques.

Intraoperative blood loss and intraoperative time were measured. After surgery, patients were shifted to the recovery room, discharged from there, and followed up for postoperative pain after 24 hours.

Both groups were prescribed the same amount of postoperative analgesia. All the data were collected through a pre-designed proforma. The collected data were entered and analyzed in Statistical Package for Social Science (SPSS) v25.0 (IBM Corp., Armonk, USA). Quantitative variables like age, BMI, duration of hemorrhoids, intraoperative blood loss, and postoperative pain were described as Mean ± S.D. Categorical variable like gender was expressed as frequencies and percentages. Data was classified for age, gender, BMI, grades, and duration of hemorrhoids. To compare the two groups regarding bleeding, postoperative pain, and operative time, an independent sample t-test was done. A p-value ≤ 0.05 was considered significant.

## Results

There were a total of 140 patients participating in this trial. In group A, there were 41 males (58.5%) and 29 females (41.4%), but in group B, there were 43 males (61.4%) and 27 females (38.5%). The p-value was 0.730, which was insignificant (Table 1). The average age of the individuals in group A was 49.97 ± 7.36 years, and in group B, it was 43.2 ± 8.01 years.

**Table 1:** Comparison of gender distribution between groups

Gender	Groups		Total
	ENSEAL®	Milligan Morgan	
Male	41	43	84
	58.5%	61.4%	60.0%
Female	29	27	56
	41.4%	38.5%	40.0%
Total	70	70	140
	100.0%	100.0%	100.0%

In group A, 54 (77.1%) had grade III hemorrhoids, while 16 (22.9%) had grade IV hemorrhoids. In group B, 50 (71.4%) had grade III hemorrhoids, while 20 (28.6%) had grade IV hemorrhoids. The p-value was 0.439, which was insignificant (Table 2)

**Table 2:** Comparison of the grade of hemorrhoids between groups

Grade of hemorrhoids	Groups		Total
	ENSEAL®	Milligan Morgan	
Grade III	54	50	104
	77.1%	71.4%	74.3%
Grade IV	16	20	36
	22.9%	28.6%	25.7%
Total	70	70	140
	100.0%	100.0%	100.0%

In group A, 50 (71.4%) had a duration of hemorrhoids of <6 months, while 20 (28.6%) had a duration of hemorrhoids of >6 months. In group B, 48 (68.6%) had a duration of hemorrhoids of <6 months, while 22 (31.4%) had a duration of hemorrhoids of >6 months (p = 0.427 i.e., insignificant). In group A, 51 (72.9%) had normal BMI, while 19 (27.1%) were overweight. In group B, 53 (75.7%) had normal BMI, while 17 (24.3%) were overweight. The p-value of BMI was 0.423, which was insignificant

In group A, the mean operating time was 20.87± 3.05 min, but in group B, it was 27.10 ± 3.42 min, which is statistically significant with a p-value of <0.001 (Table 3).

**Table 3:** Comparison of operative time between groups

Operative time (minutes)	Groups	n	Mean	Std. Deviation	p-value
	ENSEAL®	70	20.87	3.05	<0.001 Significant
	Milligan Morgan	70	27.10	3.42	

In group A, the mean blood loss was 9.79 ± 2.87 ml; however, in group B, the mean blood loss was 13.36 ± 3.73 ml, which is statistically significant with a p-value of <0.001 (Table 4).

**Table 4:** Comparison of blood loss between groups

Blood loss (ml)	Groups	n	Mean	Std. Deviation	p-value
	ENSEAL®	70	9.79	2.87	<0.001 Significant
	Milligan Morgan	70	13.36	3.34	

In group A, on a scale of 1 - 10, the mean pain score was 2.7 ± 1.08, while it was 3.34 ± 1.16 in group B, which is statistically significant with a p-value of <0.001 (Table 5).

**Table 5:** Comparison of pain between groups

Pain score	Groups	n	Mean	Std. Deviation	p-value
	ENSEAL®	70	2.7	1.08	<0.001 Significant
	Milligan Morgan	70	3.34	1.16	

## Discussion

In symptomatic hemorrhoids of grades III and IV, hemorrhoidectomy remains the standard therapy. In the absence of a superior alternative, classic approaches such as the Milligan-Morgan technique and Ferguson's technique have been utilized for over a half-century. In recent years, new strategies with respective benefits and drawbacks have emerged. A circular stapling device for prolapsed hemorrhoids is the most significant innovation of recent times. This device has been criticized for failing to cure the exterior component of hemorrhoids and skin tags. In addition, stapler cartridges are too costly for the vast majority of patients [12].

Another study was conducted in our department where a comparison was made between Milligan- Morgan technique versus the harmonic scalpel. In that study, the mean operative time was 20.8±2.8 minutes with a harmonic scalpel and 26.5±2.8 minutes with Milligan-Morgan technique [13]. These results are comparable to our study, proving ENSEAL® hemorrhoidectomy to be as efficient as harmonic scalpel haemorrhoidectomy. Kochar and Singh presented comparable results to our study, stating that the ENSEAL® hemorrhoidectomy (EH) group experienced less pain as ENSEAL® damages the nerve endings and EH has the additional benefit of ease of operating and reduced duration of operation [14].

Song et al. shared that postoperative hemorrhage is among the life-threatening complications of the Milligan-Morgan method and needs specialized care. Approximately 2-6% of individuals who undergo Milligan-Morgan experience hemorrhage postoperatively, with primary hemorrhage being more prevalent than late haemorrhage. The Milligan-Morgan haemorrhoidectomy group's incomplete excision of subsequent hemorrhoids extended postoperative perianal oedema and discomfort. In addition, postoperative perianal skin tags may develop from untreated secondary haemorrhoids, which can cause discomfort and irritation in the patient [15]. Although this study was not life-threatening, it had a high incidence of postoperative haemorrhage and pain.

Late consequences of open hemorrhoidectomy include postoperative bleeding, anal fissure, and frequent incontinence to bowel movements when using laxatives postoperatively [16]. Another recognized consequence of conventional hemorrhoidectomy, perirectal hematomas, are found in the perirectal space, within the mesorectum, in the true pelvis, and this extraperitoneal region is in connection with the mesosigma and the retroperitoneum [17].

Various modifications have been recommended in an attempt to reduce postoperative complications. Loder and Phillips became the first to describe a dissecting anatomical plane by observing short fibers extending from the internal sphincter to the anal cushions. They stressed the necessity of cutting these fibers near the anal cushions while keeping the internal sphincter undamaged. A subcutaneous fascia reaching into a membrane enclosing the internal sphincter was recognizable following the pedicle skin incision. Additionally, the knowledge of the hemorrhoidal artery supply via the rectal wall has lowered the need for pedicle ligation [16].

This study's limitations include a short follow-up period and an inability to thoroughly examine late consequences after hemorrhoidectomy, such as anal stenosis, fecal incontinence, and persistent discomfort. However, no patient in either group experienced these or any other complications.

## Conclusions

When considering the length of the procedure and blood loss, ENSEAL® hemorrhoidectomy has been determined to be an effective treatment that patients tolerate well after the procedure. Therefore, ENSEAL® hemorrhoidectomy can be a safe and efficient alternative treatment for hemorrhoids that are causing symptoms.
